# Synthesis of 2-Acyloxycyclohexylsulfonamides and Evaluation on Their Fungicidal Activity

**DOI:** 10.3390/ijms141122544

**Published:** 2013-11-14

**Authors:** Xinghai Li, Zining Cui, Xiaoyuan Chen, Decai Wu, Zhiqiu Qi, Mingshan Ji

**Affiliations:** 1 Department of Pesticide Science, Plant Protection College, Shenyang Agricultural University, Shenyang 110866, Liaoning, China; E-Mails: lixinghai30@aliyun.com (X.L.); desk101@163.com (X.C.); wudecai@163.com (D.W.); syqizhiqiu@sina.com (Z.Q.); 2 Guangdong Province Key Laboratory of Microbial Signals and Disease Control, Department of Plant Pathology, College of Natural Resources and Environment, South China Agricultural University, Guangzhou 510642, Guangdong, China; E-Mail: ziningcui@scau.edu.cn

**Keywords:** 2-acyloxycycloalkylsulfonamides, *Botrytis cinerea*, anti-fungal spectra, structure-activity relationship

## Abstract

Eighteen *N*-substituted phenyl-2-acyloxycyclohexylsulfonamides (**III**) were designed and synthesized by the reaction of *N*-substituted phenyl-2-hydroxyl-cycloalkylsulfonamides (**I**, R^1^) with acyl chloride (**II**, R^2^) in dichloromethane under the catalysis of TMEDA and molecular sieve. High fungicidal active compound *N*-(2,4,5-trichlorophenyl)-2-(2-ethoxyacetoxy) cyclohexylsulfonamide (**III-18**) was screened out. Mycelia growth assay against the *Botrytis cinerea* exhibited that EC_50_ and EC_80_ of compound **III-18** were 4.17 and 17.15 μg mL^−1^ respectively, which was better than the commercial fungicide procymidone (EC_50_ = 4.46 μg mL^−1^ and EC_80_ = 35.02 μg mL^−1^). For *in vivo* activity against *B. cinerea* in living leaf of cucumber, the control effect of compound **III-18** was better than the fungicide cyprodinil. In addition, this new compound had broader fungicidal spectra than chlorothalonil.

## 1. Introduction

**Figure 1:**
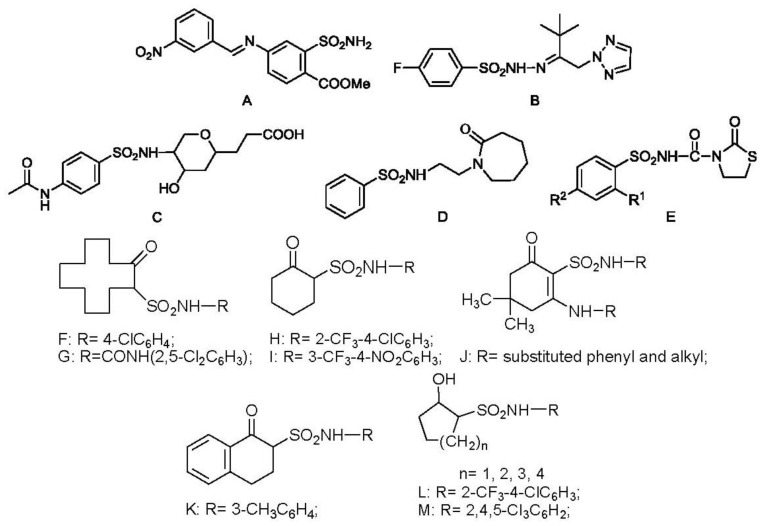
Structures of compounds **A**–**M**.

Arylsulfonamides possessed excellent bioactivity against various kinds of bacteria and fungi, which were applied as medicines [1–8] and agrichemicals [9–13]. Recently, some novel arylsulfonamides containing different scaffolds, such as benzenesulfonamide (Figure 1A) [14] and the derivatives having 1,2,3-triazol (Figure 1B) [15], pyran ring (Figure 1C) [16], 1,6-caprolactam (Figure 1D) [17] and thiazolidinone (Figure 1E) [18] were reported for their obvious and diverse fungicidal activity against *Sclerotinia sclerotiorum* (Lib.) de Bary, *Puccinia recondite* f. sp. *tritici*, *Phomopsis asparagi*, *Cladosporium fulvum* and *Fusarium oxysporum.*

To explore new potential fungicides, some novel cycloalkylsulfonamides (Figure 1F–M) were designed and synthesized in our previous works, which had very good fungicidal activity, especially against the *B. cinerea*. 2-Oxocyclododecylsulfonamides (Figure 1F) [19] and (Figure 1G) [20] showed excellent *in vitro* activity against *B. cinerea* and *Gibberella zeae* (Schw.) Petch. When the ring was contracted to six-membered ring, the compounds (Figure 1H) [21], (Figure 1I) [22] with trifluoromethyl group and compound (Figure 1J) [23] also showed strong activities against *B. cinerea* and *Sclerotinia sclerotiorum*. The derivative of tetralonesulfonamide (Figure 1K) [24] exhibited good activity against *B. cinerea* and *Rhizoctonia solani*. When the carbonyl group was changed into hydroxyl group (Figure 1L) [25,26] and (Figure 1M) [27], the activity against *B. cinerea* was improved, not only *in vitro* but also *in vivo*.

**Scheme 1:**
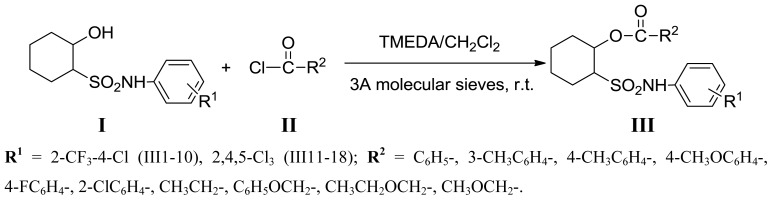
Synthetic route of compounds **III**. **R^1^** = 2-CF_3_-4-Cl (III1-10), 2,4,5-Cl_3_ (III11-18); **R^2^** = C_6_H_5_-, 3-CH_3_C_6_H_4_-, 4-CH_3_C_6_H_4_-, 4-CH_3_OC_6_H_4_-, 4-FC_6_H_4_-, 2-ClC_6_H_4_-, CH_3_CH_2_-, C_6_H_5_OCH_2_-, CH_3_CH_2_OCH_2_-, CH_3_OCH_2_-.

The ester group is the common bioactive moiety and 2-hydroxycycloalkylsulfonamides possess promising fungicidal activity. In continuation of our research on the synthesis of biological cycloalkylsulfonamide compounds, esterification was applied to the *N*-substituted phenyl-2-hydroxycycloalkylsulfonamides (**I**), which was as the lead compounds to get the title compounds **III** (Scheme 1). Their fungicidal activity against *B. cinerea* and other 10 kinds of pathogenic fungi were evaluated to obtain the optimized compounds with significantly improved fungicidal activity and broad antifungal spectra.

## 2. Results and Discussion

### 2.1. Synthesis and Structure Elucidation

Oxalyl chloride was used as the chlorinating agent in this experiment, which was reacted rapidly under the catalysis of DMF. The reaction made a very high yield of the acyl chloride, which was reacted with the compounds containing hydroxy group under the catalysis of TMDEA and molecular sieves to obtain a very high yield of target compounds.

**Figure 2:**
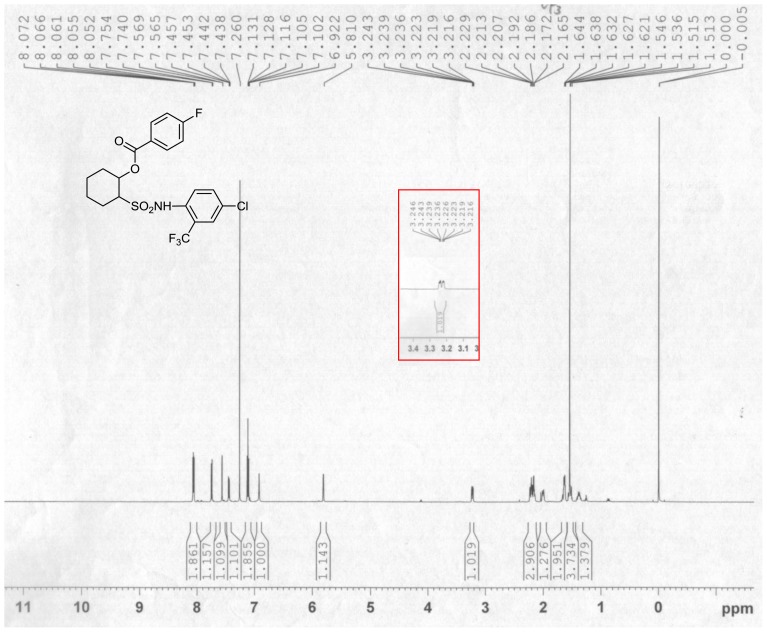
^1^H-NMR spectrum of the compound **III**-**5**.

**Figure 3:**
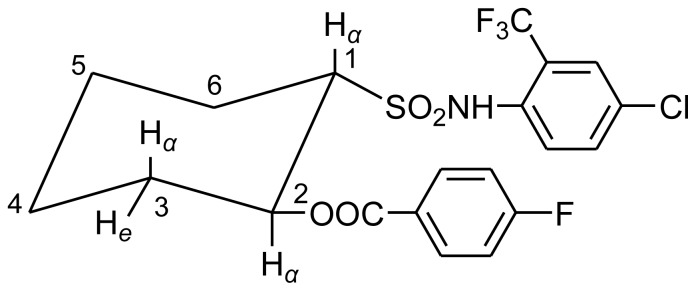
Proposed conformation of the compound **III**-**5**.

In the ^1^H NMR spectra of the compound **III-5**, 2-H_α_ was split by 1-H_α_, 3-H_e_ and 3-H_α_ in the cyclohexane, forming the splitting ddd peak (Figure 2). It may be the *trans* chair-conformation in Figure 3. Due to the directly induced effect of the sulfonyl group and the *ortho*-position effect of the acyloxy to the 1-H_α_, which made the CH signal slightly wide single peak in the spectra, the peak type was similar to that of NH in sulfonamide structure. In the IR spectrum, the very obvious ester carbonyl stretching vibration appeared around 1700 cm^−1^. NH stretching vibration was around 3200 cm^−1^ to 3500 cm^−1^.

### 2.2. Screening of Fungicidal Activity and SAR

#### 2.2.1. *In Vitro* Fungicidal Activity of Compounds **III-1**–**III-18** against *B. cinerea*

From the results of mycelial growth rate (Table 1) and structure-activity relationship (SAR), it could be seen that acyloxy substituent showed higher antifungal activity when it was a small group. Among them, 2-substituted benzoyloxy compounds showed generally lower activity, while 2-(2-alkoxyacetyloxy) cycloalkylsulfonamides showed good activity. The same structure-activity relationship appeared in the test of detached leaves of cucumber (Table 1). It showed obvious bioactivity when the R^2^ was ethoxymethyl and methoxymethyl. The bioactivity of the *N*-(2,4,5-trichlorophenyl)-2-acyloxycyclohexylsulfonamides were better than that of the *N*-(2-trifluoromethyl-4-chlorinephenyl)-2-acyloxycyclohexyl-sulfonamides. The fungicidal activity of **III-18** was a little higher than that of the procymidone *in vitro* tests. While the detached leaves test showed that the control efficiency of **III-18** was significantly higher than that of the procymidone. According to Regulation (EC) No 1107/2009, procymidone is no longer approved in the European Union. Because of the great fungicidal activity, **III-18** could be a potential substitute of this fungicide.

#### 2.2.2. Fungicidal Activity of Compounds **III-9** and **III-18** against Ten Pathogenic Fungi

The screening data in Table 2 indicated that the two compounds **III**-**9** and **III**-**18** had broad fungicidal spectra. **III**-**9** exhibited moderate activity against ten pathogenic fungi. Compound **III**-**18** exhibited excellent bioactivity against the most of the selected fungus species (except for the *R. solani*) and the inhibitory rate was similar to or better than the commercialized fungicide chlorothalonil.

#### 2.2.3. *In Vivo* Fungicidal Activity of Compounds **III-18**, **19** and **20** against *B. cinerea*

**Figure 4:**
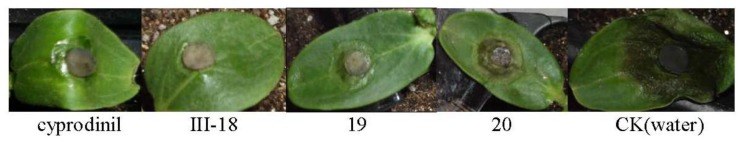
*In vivo* activity against *B. cinerea* in cucumber seedlings at 125 μg mL^−1^.

In addition, the concentration gradient experiment was evaluated by the method of cucumber leaf to check the fungicidal activity of compounds **III-18**, *N*-(2,4,5-trichlorophenyl)-2-hydroxycycloalkylsulfonamide (**19**) and *N*-(2,4,5-trichlorophenyl)-2-oxycycloalkylsulfonamide (**20**). The results in Table 3 showed that the activity of the compounds was gradually improved after continuous structural optimization. From the results, it could be seen that the bioactivity was not obviously different between **III-18** and **19**, which was much better than the activity of **20**, and a little better than that of the control cyprodinil. The pathogen infection symptoms (Figure 4) showed that the leaves treated by the compound **III-18** were still green and the disease spots were wet blotch plaque, which was better than the leaves treated by other compounds. The leaves treated by **20** and blank control were black and rotten.

## 3. Experimental Section

### 3.1. General Information

Infrared (IR) spectra were recorded in potassium bromide disks on a Perkin Elmer Spectrum 65 spectrophotometer. Nuclear magnetic resonance (NMR) spectra were recorded in CDCl_3_ unless indicated otherwise with Bruker 600 MHz or 300 MHz spectrometers, using tetramethylsilane (TMS) as the internal standard. Elemental analysis was performed by the analytical center at the Institute of Chemistry, Chinese Academy of Sciences, Beijing, China. Melting points were measured on an X-5 melting-point apparatus, and the thermometer was uncorrected. The solvents and reagents were used as received or were dried prior to use as needed.

### 3.2. Synthetic Procedures

#### 3.2.1. Synthesis of Compounds **I**

Compounds **I** were synthesized according to the method given in the references [25] and [27].

#### 3.2.2. Synthesis of Acyl Chloride **II**

To the solution of acid (0.05 mol) and *N*,*N*-dimethyl formamide (DMF, 0.05 mL) in dry dichloromethane (20 mL), oxalyl chloride (0.06 mol) was added dropwise. The mixture was stirred at 20–25 °C for 4 h. And the acyl chloride was distilled under pressure.

**II-1**: Benzoyl chloride, colorless liquid, bp 91–92 °C/31 mmHg, yield 85%.
**II-2**: 3-Methylbenzoyl chloride, colorless liquid, bp 90–92 °C/10 mmHg, yield 86%.
**II-3**: 4-Methylbenzoyl chloride, colorless liquid, bp 117–119 °C/15 mmHg, yield 88%.
**II-4**: 4-Methoxybenzoyl chloride, white solid, bp 153–156 °C/15mmHg, yield 80%.
**II-5**: 4-Fluorobenzoyl chloride, colorless liquid, bp 87–88 °C/25 mmHg, yield 88%.
**II-6**: 2-Chlorobenzoyl chloride, colorless liquid, bp 110–114 °C/8 mmHg, yield 85%.
**II-7**: Propionyl chloride, colorless, bp 77–79 °C, yield 84%.
**II-8**: Phenoxyacetyl chloride, colorless liquid, bp 103–105 °C/7 mmHg, yield 82%.
**II-9**: 2-Ethoxyacetyl chloride, colorless liquid, bp 65–67 °C/33 mmHg, yield 82%.
**II-10**: 2-Methoxyacetyl chloride, colorless liquid, bp 112–113 °C, yield 85%.

#### 3.2.3. Synthesis of Compounds **III-1**–**III-18**

According to the method given in the reference [28], to the solution of **I** (0.01 mol), *N*,*N*,*N*′,*N*′-tetramethylethylenediamine (TMEDA, 0.006 mol) and 3A molecular sieves (2 g) in dry dichloromethane (30 mL), acyl chloride **II** (0.011 mol) was added dropwise at room temperature. The mixture was stirred at room temperature for 2 h. And the reaction was quenched by ice water (20 mL × 2), filtered and dried with anhydrous magnesium sulfate. After evaporating the solvent under vacuum, the crude product was purified by silica gel chromatography using petroleum/ethyl acetate (10/1, *v*/*v*) as eluant to obtain **III** (Scheme 1).

##### N-(2-Trifluoromethyl-4-chlorophenyl)-2-benzoyloxy-cyclohexylsulfonamide (**III-1**)

White crystal, yield 90%, m.p. 119–121 °C, ^1^H-NMR (CDCl_3_, 300 MHz, Figure S1) δ: 1.37–2.25 (m, 8H, 4CH_2_), 3.22–3.28 (m, 1H, CH–O), 5.84 (s, 1H, CH–SO_2_), 6.98 (s, 1H, NH), 7.38–8.06 (m, 8H, C_6_H_3_ + C_6_H_5_); IR(KBr) ν: 3273, 2944, 2858, 1717 cm^−1^. Anal calcd. for C_20_H_19_ClF_3_NO_4_S: C 52.01, H 4.15, N 3.03; Found: C 51.82, H 4.13, N, 3.21.

##### N-(2-Trifluoromethyl-4-chlorophenyl)-2-(3-methyl-benzoyloxy)-cyclohexylsulfonamide (**III-2**)

White crystal, yield 88%, m.p. 112–114 °C, ^1^H-NMR (CDCl_3_, 300 MHz, Figure S2) δ : 1.41–2.25 (m, 8H, 4CH_2_), 2.40 (s, 3H, CH_3_), 3.22–3.27 (m, 1H, CH–O), 5.83 (s, 1H, CH–SO_2_), 6.98 (s, 1H, NH), 7.29–7.83 (m, 7H, C_6_H_3_ + C_6_H_4_); IR(KBr) ν: 3283, 2939, 2855, 1720 cm^−1^. Anal calcd. for C_21_H_21_ClF_3_NO_4_S: C 53.00, H 4.45, N 2.94; Found: C 53.41, H 4.28, N 2.96.

##### N-(2-Trifluoromethyl-4-chlorophenyl)-2-(4-methyl-benzoyloxy)-cyclohexylsulfonamide (**III-3**)

White crystal, yield 87%, m.p. 128–130 °C, ^1^H-NMR (CDCl_3_, 600 MHz, Figure S3) δ : 1.37–2.23 (m, 8H, 4CH_2_), 2.40 (s, 3H, CH_3_), 3.22–3.24 (m, 1H, CH–O), 5.81 (s, 1H, CH–SO_2_), 6.95 (s, 1H, NH), 7.22–7.91 (m, 7H, C_6_H_3_ + C_6_H_4_); IR(KBr) ν: 3277, 2949, 2865, 1708 cm^−1^. Anal calcd. for C_21_H_21_ClF_3_NO_4_S: C 53.00, H 4.45, N 2.94; Found: C 52.97, H 4.50, N 2.91.

##### N-(2-Trifluoromethyl-4-chlorophenyl)-2-(4-methoxy-benzoyloxy)-cyclohexylsulfonamide (**III-4**)

White crystal, yield 83%, m.p. 94–96 °C, ^1^H-NMR (CDCl_3_, 600 MHz, Figure S4) δ : 1.37–2.22 (m, 8H 4CH_2_), 3.21–3.24 (m, 1H CH–O), 3.86 (s, 3H OCH_3_), 5.79 (s, 1H CH–SO_2_), 6.98 (s, 1H NH), 6.90–7.99 (m, 7H C_6_H_3_ + C_6_H_4_); IR(KBr) ν: 3277, 2949, 2865, 1708 cm^−1^. Anal calcd. for C_21_H_21_ClF_3_NO_5_S: C 51.27; H 4.30, N 2.85; Found: C 51.21; H 4.19, N 2.93.

##### N-(2-Trifluoromethyl-4-chlorophenyl)-2-(4-fluoro-benzoyloxy)-cyclohexylsulfonamide (**III-5)**

White crystal, yield 89%, m.p. 120–122 °C, ^1^H-NMR (CDCl_3_, 600 MHz, Figure S5) δ : 1.37–2.22 (m, 8H, 4CH_2_), 3.23 (ddd, *J* = 12, 4.2, 1.8 Hz, 1H, CH–O), 5.81 (s, 1H, CH–SO_2_), 6.92 (s, 1H, NH), 7.09–8.07 (m, 7H, C_6_H_3_ + C_6_H_4_); IR(KBr) ν: 3277, 2935, 2859, 1760 cm^−1^. Anal calcd. for C_20_H_18_ClF_4_NO_4_S: C 50.06, H 3.78, N 2.92; Found: C 50.05, H 3.89, N 2.79.

##### N-(2-Trifluoromethyl-4-chlorophenyl)-2-(2-chloro-benzoyloxy)-cyclohexylsulfonamide (**III-6**)

White crystal, yield 86%, m.p. 98–100 °C, ^1^H-NMR (CDCl_3_, 600 MHz, Figure S6) δ: 1.37–2.25 (m, 8H, 4CH_2_), 3.21–3.24 (m, 1H, CH–O), 5.81 (s, 1H, CH–SO_2_), 6.96 (s, 1H, NH), 7.31–7.93 (m, 7H, C_6_H_3_ + C_6_H_4_); IR(KBr) ν: 3247, 2954, 2869, 1731 cm^−1^. Anal calcd. for C_20_H_18_Cl_2_F_3_NO_4_S: C 48.40, H 3.66, N 2.82; Found: C 48.20, H 3.45, N 2.92.

##### N-(2-Trifluoromethyl-4-chlorophenyl)-2-propionyloxy-cyclohexylsulfonamide (**III-7**)

Oil, yield 73%, ^1^H-NMR (CDCl_3_, 600 MHz, Figure S7) δ: 1.13–2.41 (m, 13H, 4CH_2_ + C_2_H_5_), 3.08–3.11 (m, 1H, CH–O), 5.66 (s, 1H, CH–SO_2_), 7.06 (s, 1H, NH), 7.49–7.72 (m, 3H, C_6_H_3_); IR(KBr) ν: 3276, 2959, 2859, 1752 cm^−1^. Anal calcd. for C_16_H_19_ClF_3_NO_4_S: C 46.44, H 4.63, N 3.38; Found: C 46.22, H 4.43, N 3.54.

##### N-(2-Trifluoromethyl-4-chlorophenyl)-2-phenoxyacetoxy-cyclohexylsulfonamide (**III-8**)

Oil, yield 78%, ^1^H-NMR (CDCl_3_, 600 MHz, Figure S8) δ: 1.13–2.05 (m, 8H, 4CH_2_), 3.09–3.12 (m, 1H, CH–O), 4.70 (s, 2H, CH_2_–O), 5.77 (s, 1H, CH–SO_2_), 6.88–7.71 (m, 9H, C_6_H_3_ + C_6_H_5_ + NH); IR(KBr) ν: 3278, 2944, 2864, 1754 cm^−1^. Anal calcd. for C_21_H_21_ClF_3_NO_5_S: C 51.27, H 4.30, N 2.85; Found: C 51.43, H 4.38, N 2.65.

##### N-(2-Trifluoromethyl-4-chlorophenyl)-2-(2-ethoxyacetoxy)-cyclohexylsulfonamide (**III-9**)

White crystal, yield 79%, m.p. 85–87 °C, ^1^H-NMR (CDCl_3_, 600 MHz, Figure S9) δ: 1.22–2.09 (m, 11H, 4CH_2_ + CH_3_), 3.10–3.13 (m, 1H, CH–O), 3.62 (q, *J* = 7.2 Hz, 2H, CH_2_O), 4.12 (s, 2H, OCH_2_CO), 5.74 (s, 1H, CH–SO_2_), 7.02 (s, 1H, NH), 7.50–7.71 (m, 3H, C_6_H_3_); IR(KBr) ν: 3282, 2959, 2859, 1760 cm^−1^. Anal calcd. for C_17_H_21_ClF_3_NO_5_S: C 46.00, H 4.77, N 3.16; Found: C 45.87, H 4.79, N 3.32.

##### N-(2-Trifluoromethyl-4-chlorophenyl)-2-(2-methoxyacetoxy)-cyclohexylsulfonamide (**III-10**)

White crystal, yield 83%, m.p. 94–96 °C, ^1^H-NMR (CDCl_3_, 300 MHz, Figure S10) δ: 1.33–2.08 (m, 8H, 4CH_2_), 3.09–3.15 (m, 1H, CH–O), 3.47 (s, 3H, CH_3_O), 4.08 (s, 2H, OCH_2_CO), 5.75 (s, 1H, CH–SO_2_), 7.03 (s, 1H, NH), 7.49–7.72 (m, 3H, C_6_H_3_); IR(KBr) ν: 3278, 2953, 2860, 1751 cm^−1^. Anal calcd. for C_16_H_19_ClF_3_NO_5_S: C 44.71, H 4.46, N 3.26; Found: C 44.55, H 4.52, N 3.19.

##### N-(2,4,5-Trichlorophenyl)-2-benzoyloxy-cyclohexylsulfonamide (**III-11**)

White crystal, yield 76%, m.p. 179–181 °C, ^1^H-NMR (CDCl_3_, 300 MHz, Figure S11) *δ* (ppm): 1.41–2.27 (m, 8H, 4CH_2_), 3.22–3.28 (m, 1H, CH–O), 5.83 (s, 1H, CH–SO_2_), 7.39–8.07 (m, 8H, C_6_H_2_ + C_6_H_5_ + NH); IR(KBr) ν: 3236, 2946, 2859, 1761 cm^−1^. Anal calcd. for C_19_H_18_Cl_3_NO_4_S: C 49.31, H 3.92, N 3.03; Found: C 49.69, H 3.79, N 3.05.

##### N-(2,4,5-Trichlorophenyl)-2-(3-methylbenzoyloxy)-cyclohexylsulfonamide (**III-12**)

White crystal, yield 78%, m.p. 176–178 °C, ^1^H-NMR (CDCl_3_, 300 MHz, Figure S12) δ: 1.45–2.27 (m, 8H, 4CH_2_), 2.41 (s, 3H, CH_3_), 3.18–3.24 (m, 1H, CH–O), 5.83 (s, 1H, CH–SO_2_), 7.20 (s, 1H, NH), 7.29–7.83 (m, 6H, C_6_H_2_ + C_6_H_4_); IR(KBr) ν: 3280, 2940, 2854, 1754 cm^−1^. Anal calcd. for C_20_H_20_Cl_3_NO_4_S: C 50.38, H 4.23, N 2.94; Found: C 50.25, H 4.08, N 2.99.

##### N-(2,4,5-Trichlorophenyl)-2-(4-methylbenzoyloxy)-cyclohexylsulfonamide (**III-13**)

White crystal, yield 78%, m.p. 170–172 °C, ^1^H-NMR (CDCl_3_, 600 MHz, Figure S13) δ: 1.42–2.37 (m, 8H, 4CH_2_), 2.41 (s, 3H, CH_3_), 3.19–3.22 (m, 1H, CH–O), 5.83 (d,d,d, *J* = 11.70, 3.90, 1.80 Hz, 1H, CH-SO_2_), 7.18 (s, 1H, NH), 7.30–7.83 (m, 6H, C_6_H_2_ + C_6_H_4_); IR(KBr) ν: 3278, 2946, 2859, 1708 cm^−1^. Anal calcd. for C_20_H_20_Cl_3_NO_4_S: C 50.38, H 4.23, N 2.94; Found: C 50.63, H 4.11, N 2.97.

##### N-(2,4,5-Trichlorophenyl)-2-(4-fluorobenzoyloxy)-cyclohexylsulfonamide (**III-14**)

White crystal, yield 79%, m.p. 160–161 °C, ^1^H-NMR (CDCl_3_, 300 MHz, Figure S14) δ: 1.49–2.25 (m, 8H, 4CH_2_), 3.15–3.20 (m, 1H, CH–O), 5.89 (s, 1H, CH–SO_2_), 7.20 (s, 1H, NH), 7.29–7.90 (m, 6H, C_6_H_2_ + C_6_H_4_); IR(KBr) ν: 3278, 2940, 2859, 1708 cm^−1^. Anal calcd. for C_19_H_17_Cl_3_FNO_4_S: C 47.47, H 3.56, N 2.91; Found: C 46.99, H 3.45, N 2.97.

##### N-(2,4,5-Trichlorophenyl)-2-(2-chlorobenzoyloxy)-cyclohexylsulfonamide (**III-15**)

White crystal, yield 75%, m.p. 145–147 °C, ^1^H-NMR (CDCl_3_, 300 MHz, Figure S15) δ: 1.43–2.25 (m, 8H, 4CH_2_), 3.16–3.21 (m, 1H, CH–O), 5.81 (s, 1H, CH–SO_2_), 7.94–8.90 (m, 7H, C_6_H_2_ + C_6_H_4_ + NH); IR(KBr) ν: 3244, 2946, 2859, 1708 cm^−1^. Anal calcd. for C_19_H_17_Cl_4_NO_4_S: C 45.90, H 3.45, N 2.82; Found: C 46.23, H 3.44, N 2.76.

##### N-(2,4,5-Trichlorophenyl)-2-propionyloxy-cyclohexylsulfonamide (**III-16**)

White crystal, yield 56%, m.p. 130–131 °C, ^1^H-NMR (CDCl_3_, 600 MHz, Figure S16) δ: 1.17–2.43 (m, 13H, 4CH_2_ + C_2_H_5_), 3.01–3.04 (m, 1H, CH–O), 5.65 (s, 1H, CH–SO_2_), 7.32 (s, 1H, NH), 7.50–7.82 (m, 2H, C_6_H_2_); IR(KBr) ν: 3251, 2940, 2859, 1734 cm^−1^. Anal calcd. for C_15_H_18_Cl_3_NO_4_S: C 43.44, H 4.37, N 3.38; Found: C 43.55, H 4.49, N 3.31.

##### N-(2,4,5-Trichlorophenyl)-2-phenoxyacetoxy-cyclohexylsulfonamide (**III-17**)

White crystal, yield 55%, m.p. 146–148 °C, ^1^H-NMR (CDCl_3_, 600 MHz, Figure S17), *δ* (ppm): 1.31–2.13 (m, 8H, 4CH_2_), 3.02–3.06 (m, 1H, CH–O), 4.71 (s, 2H, CH_2_-O), 5.77 (s, 1H, CH–SO_2_), 6.95–7.83 (m, 8H, C_6_H_2_ + C_6_H_5_ + NH); IR(KBr) ν: 3317, 2940, 2859, 1728 cm^−1^. Anal calcd. for C_20_H_20_Cl_3_NO_5_S: C 48.74, H 4.09, N 2.84; Found: C 48.66, H 4.01, N 2.98.

##### N-(2,4,5-Trichlorophenyl)-2-(2-ethoxyacetoxy)-cyclohexylsulfonamide (**III-18**)

White crystal, yield 43%, m.p. 124–126 °C, ^1^H-NMR (CDCl_3_, 600 MHz, Figure S18) δ: 1.24–2.12 (m, 11H, 4CH_2_ + CH_3_), 3.03–3.06 (m, 1H, CH–O), 3.63 (q, *J* = 7.2 Hz, 2H, CH_2_O), 4.15 (s, 2H, OCH_2_CO), 5.73 (s, 1H, CH–SO_2_), 7.34 (s, 1H, NH), 7.49–7.84 (m, 2H, C_6_H_2_); IR(KBr) ν: 3336, 2940, 2859, 1754 cm^−1^. Anal calcd. for C_16_H_20_Cl_3_NO_5_S: C 43.21, H 4.53, N 3.15; Found: C 42.86, H 4.46, N 3.21.

### 3.3. Bioassay of Fungicidal Activity

#### 3.3.1. Effect of the Title Compounds on the Mycelial Growth

Fungicidal activities of the compounds against *B. cinerea* were evaluated using the method given in reference [27]. The title compounds **III** were dissolved in acetone and mixed with sterile molten potato dextrose agar (PDA) to obtain the gradient concentrations of 50, 25, 12.5, 6.25, 3.13, 1.56 and 0.78 μg mL^−1^. The commercial fungicide procymidone (with a percentage composition of 96%) was used as the positive control. The inhibition rate was calculated according to Equation (1). The EC_50_ and EC_80_ values of compounds **III** were estimated using logit analysis, and the results are given in Table 1.

(1) $$I_{1}=({\bar{D}}_{1}-{\bar{D}}_{0})/{\bar{D}}_{1}\times 100\%$$

where *I*_1_ is the inhibition rate, *D*_1_ is the average diameter of mycelia in the blank test, and *D*_0_ is the average diameter of mycelia in the presence of compounds.

#### 3.3.2. Effect of Compounds **III-9** and **III-18** against Ten Major Crop Diseases

The fungicidal activity of compounds **III-9** and **III-18** against ten kinds of fungi was assessed using the mycelium growth test on PDA. The compounds were dissolved in acetone and mixed PDA to obtain final concentration of 50 μg mL^−1^. Chlorothalonil was used as the positive control. Other test conditions were the same with the method given in the mycelial growth, and the results were shown in Table 2.

#### 3.3.3. Effect on the Ability of *B. cinerea* to Colonize Detached Leaves of Cucumber

The compounds **III-1**–**III-18** were confected to 2.5% EC formulations, which were diluted to concentration of 500 μg mL^−1^ with water to obtain the solutions that were spread on the surface of the cucumber leaves. After air drying for 2 h at 23 °C, the upper sides of the leaves were inoculated with 5 mm plugs of *B. cinerea*, which was maintained on PDA [25]. The cucumber leaves were maintained at 24 ± 1 °C in culture dishes. The inhibition rate was calculated and the results were shown in Table 1.

#### 3.3.4. *In Vivo* Fungicidal Activity against *B. cinerea* by Pot Culture Test Method in Greenhouse

Using pot culture test method according to the reference [25], the *in vivo* fungicidal activity of the title compounds against *B. cinerea* was evaluated in greenhouse. *B. cinerea* was maintained on potato dextrose agar (PDA) medium at 4 °C. The culture plates were cultivated at 24 ± 1 °C. Germination was conducted by soaking cucumber seeds in water for 2 h at 50 °C and then keeping the seeds moist for 24 h at 28 °C in an incubator. When the radicles were 0.5 cm, the seeds were grown in plastic pots containing a 1:1 (*v*/*v*) mixture of vermiculite and peat. Cucumber plants used for inoculations were at the stage of two seed leaves. The compounds **III-18**, **19 and 20** were confected to 2.5% EC formulations, which were diluted to concentration of 500, 125 and 31.25 μg mL^−1^ with water to obtain the solutions. Tested compounds and commercial fungicides were sprayed with a hand spray on the surface of the seed leaves. Water sprayed seed leaves were set as the CK (Figure 4). After drying, the upper sides of the leaves were inoculated with 5 mm plugs of *B. cinerea*, which was maintained on PDA [25]. The plants were maintained at 24 ± 1 °C and above 80% relative humidity in greenhouse. The fungicidal activity was evaluated and the results were shown in Table 3 and Figure 4.

## 4. Conclusions

*N*-substituted phenyl-2-acyloxycyclohexylsulfonamides were designed and synthesized. Their structures were confirmed by ^1^H NMR, IR and elementary analysis. Their fungicidal activity was better than the leading compounds *N*-substituted phenyl-2-hydroxycycloalkylsulfonamides. Among them, compound **III-18** showed better *in vitro* and *in vivo* fungicidal activity than the fungicides procymidone and cyprodinil. In addition, this new compound had broader fungicidal spectra than chlorothalonil. The preliminary structure and activity relationship showed that the 2-alkoxyacetoxy groups were considerably more likely to improve the activity of these compounds.

## Supplementary Information



## Figures and Tables

**Table 1:** Fungicidal activity of compounds **III-1**–**III-18** against *B. cinerea*.

Compd.	Mycelial growth rate method		Control efficiency on detached leaves of cucumber (500 μg mL^−1^) (%) (±SEM)

	EC_50_ (95% confidence limit)/(μg mL^−1^)	EC_80_ (95% confidence limit)/(μg mL^−1^)	
**III-1**	24.36 (10.23–57.99)	>100	61.11 (±5.73) de
**III-2**	19.94 (10.51–37.80)	>100	27.35 (±2.65) kl
**III-3**	>100	>100	53.33 (±1.20) fg
**III-4**	72.28 (7.10–735.67)	>100	18.67 (±3.03) kl
**III-5**	79.66 (7.90–802.80)	>100	43.33 (±8.89) ghi
**III-6**	150.33 (50.71–445.65)	>100	58.67 (±6.97) defg
**III-7**	6.35 (5.33–7.57)	16.72 (14.03–19.92)	57.58 (±3.50) fg
**III-8**	56.23 (18.13–173.76)	>100	48.81 (±9.20) gh
**III-9**	7.98 (6.71–9.48)	20.78 (17.49–24.69)	80.94 (±2.98) ab
**III-10**	7.46 (5.75–9.69)	31.31 (24.13–40.68)	70.67 (±8.70) cde
**III-11**	>100	-	43.60 (±3.25) hij
**III-12**	>100	-	39.74 (±7.32) jkl
**III-13**	23.86 (16.65–34.19)	88.48 (61.75–126.78)	40.47 (±9.55) ij
**III-14**	6.20 (5.08–7.58)	14.37 (11.77–17.55)	45.75 (±12.55) hi
**III-15**	29.05 (9.59–87.99)	>100	60.46 (±9.98) efg
**III-16**	5.40 (3.66–7.96)	23.13 (15.69–34.11)	58.01 (±10.22) gh
**III-17**	16.13 (11.02–23.58)	92.93 (63.55–135.90)	50.30 (±6.69) ghi
**III-18**	4.17 (3.26–5.33)	17.15 (13.41–21.94)	96.22 (±13.10) a
**procymidone**	4.46 (2.28–8.76)	35.02 (17.94–68.78)	72.11 (±5.60) bc

The letters a–l denoted the results of difference significance analysis. Means followed by the same letter within the same column are not significantly different (*p* ≥ 0.05, Fisher’s LSD multiple comparison test).

**Table 2:** Fungicidal activity of compounds **III-9** and **III-18** against ten pathogenic fungi.

Fungus	Inhibitory rate (50 μg mL^−1^) (%) (±SEM)		

	III-9	III-18	chlorothalonil
*Curvularia lunata* (Walker) Boed	66.78 (±3.07)	82.93 (±1.58)	86.39 (±2.05)
*Bipolaris maydis* Shoem	58.05 (±5.38)	87.99 (±1.25)	90.00 (±1.38)
*Gibberella zeae* (Schw.) Petch	59.01 (±1.48)	73.23 (±1.80)	90.96 (±1.48)
*Colletotrichum gossypii* Southw	75.53 (±1.87)	74.42 (±1.01)	51.73 (±1.57)
*Sclerotinia sclerotiorum* (Lib.) de Bary	78.13 (±0.90)	73.13 (±1.28)	95.99 (±0.49)
*Fusarium oxysporum*	24.02 (±2.13)	49.77 (±1.40)	78.07 (±1.35)
*Macrophoma kuwatsukai* Hara	53.12 (±1.23)	84.91 (±2.40)	79.61 (±2.35)
*Alternaria solani* Jones et Grout	53.76 (±2.30)	77.03 (±1.45)	58.94 (±5.17)
*Corynespora cassiicola*	48.9 (±1.10)	77.77 (±3.83)	69.52 (±3.26)
*Rhizoctonia solani* kühn	19.64 (±3.88)	44.13 (±2.91)	68.65 (±2.26)

**Table 3:** Control efficiency of **III-18**, **19 and 20** against *B. cinerea* (leaf method).

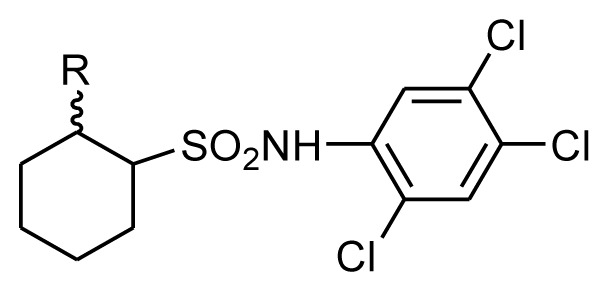				

Concentration (μg mL^−1^)	Compd.	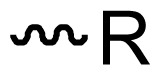	Average diameter of the spot (mm) (±SEM)	Inhibitory rate (%)
500	**III-18**	−OCOCH_2_OC_2_H_5_	3.0 (±0.6)	84.12 a
	**19**	−OH	4.3 (±0.5)	77.40 a
	**20**	=O	7.0 (±1.9)	63.21 b
	cyprodinil		4.1 (±0.5)	78.47 a
125	**III-18**	−OCOCH_2_OC_2_H_5_	5.4 (±0.6)	71.50 a′
	**19**	−OH	6.7 (±0.9)	64.40 a′b′
	**20**	=O	8.5 (±1.2)	55.13 b′
	cyprodinil		7.0 (±0.8)	62.80 b′
31.25	**III-18**	−OCOCH_2_OC_2_H_5_	9.6 (±1.4)	49.21 a″
	**19**	−OH	10.5 (±2.0)	44.22 a″
	**20**	=O	9.1 (±1.8)	52.02 a″
	cyprodinil		8.7 (±0.9)	54.12 a″
0	CK		18.9 (±2.3)	2.29

The letters a–a″ denoted the results of difference significance analysis. Means followed by the same letter within the same column are not significantly different (*p ≥* 0.05, Fisher’s LSD multiple comparison test).
