# Supplementary material for: Synthesis of 2-Acyloxycyclohexylsulfonamides and Evaluation on Their Fungicidal Activity
Source: Int J Mol Sci. 2013 Nov 14;14(11):22544–57. doi: 10.3390/ijms141122544 (PMC3856078; doi:10.3390/ijms141122544)

Supplementary Information

Figure S1. ^{1}H-NMR spectrum of compound **III-1**.

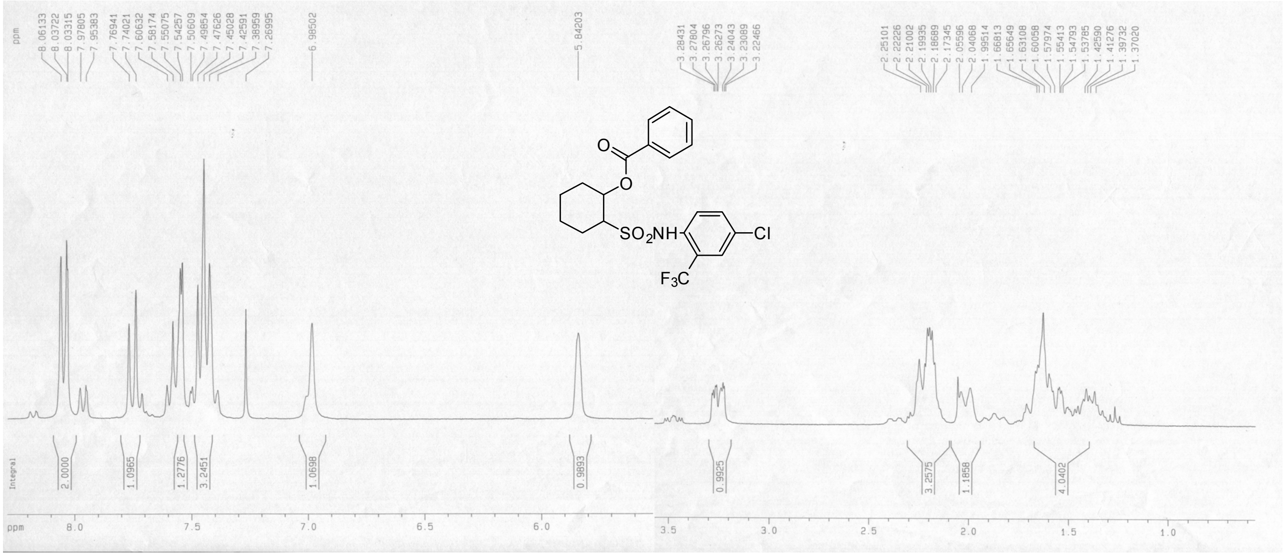

Figure S2. ^{1}H-NMR spectrum of compound **III-2**.

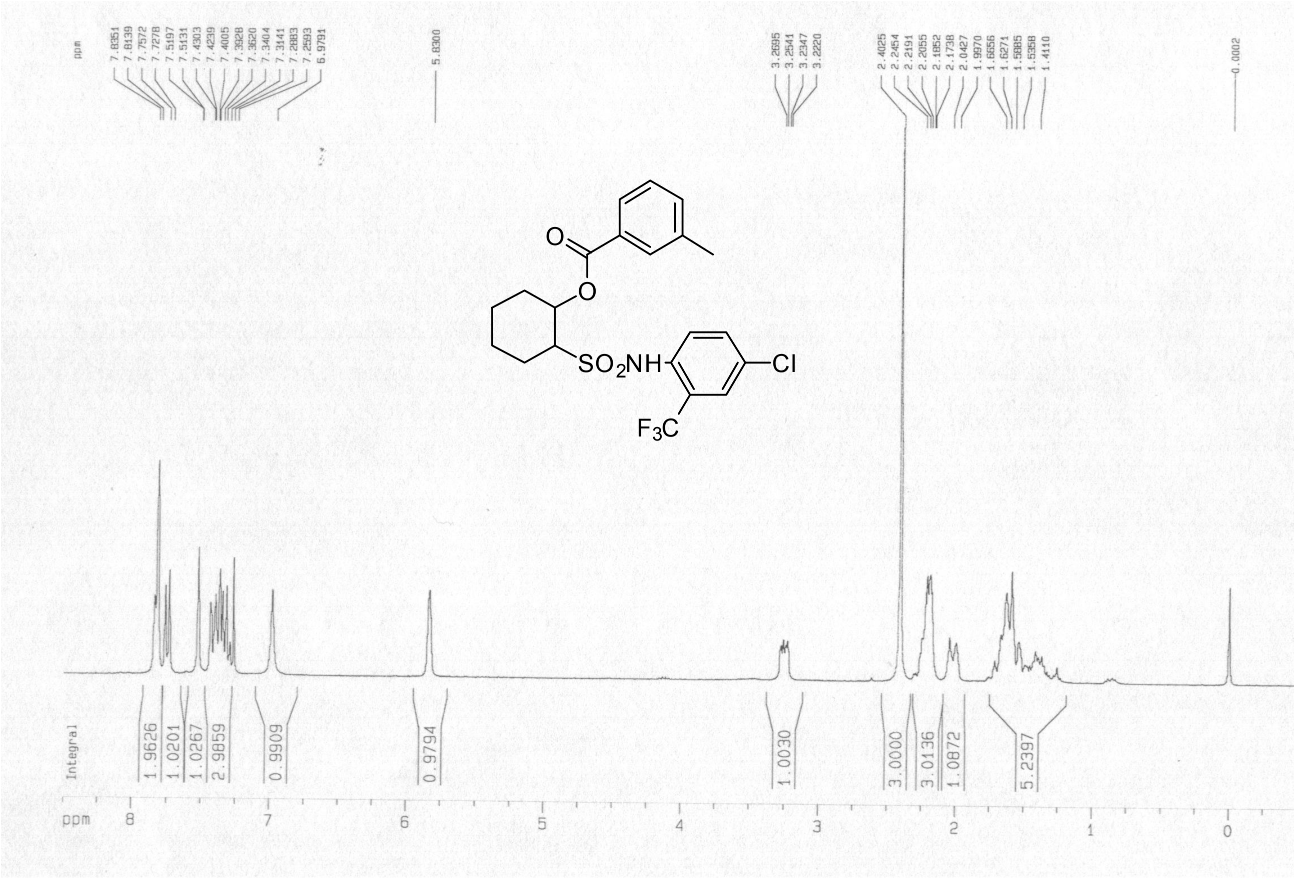

Figure S3. ^{1}H-NMR spectrum of compound **III-3**.

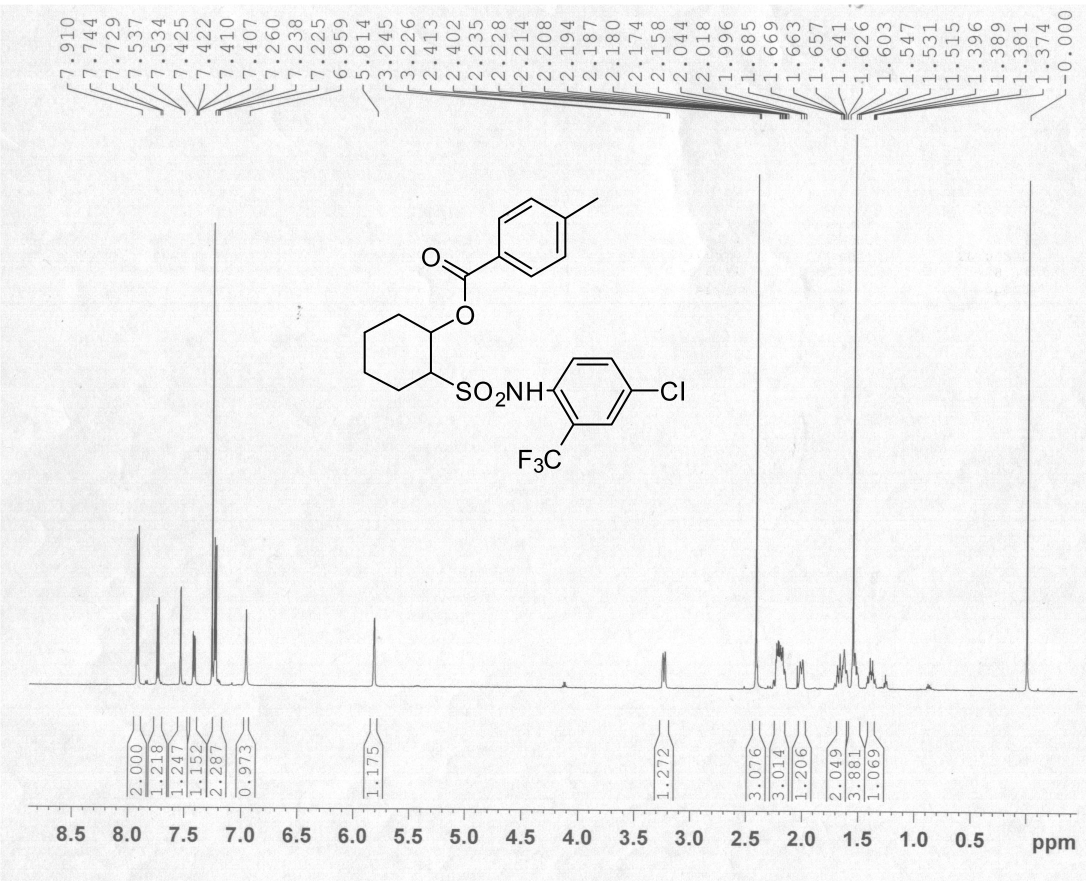

Figure S4. ^{1}H-NMR spectrum of compound **III-4**.

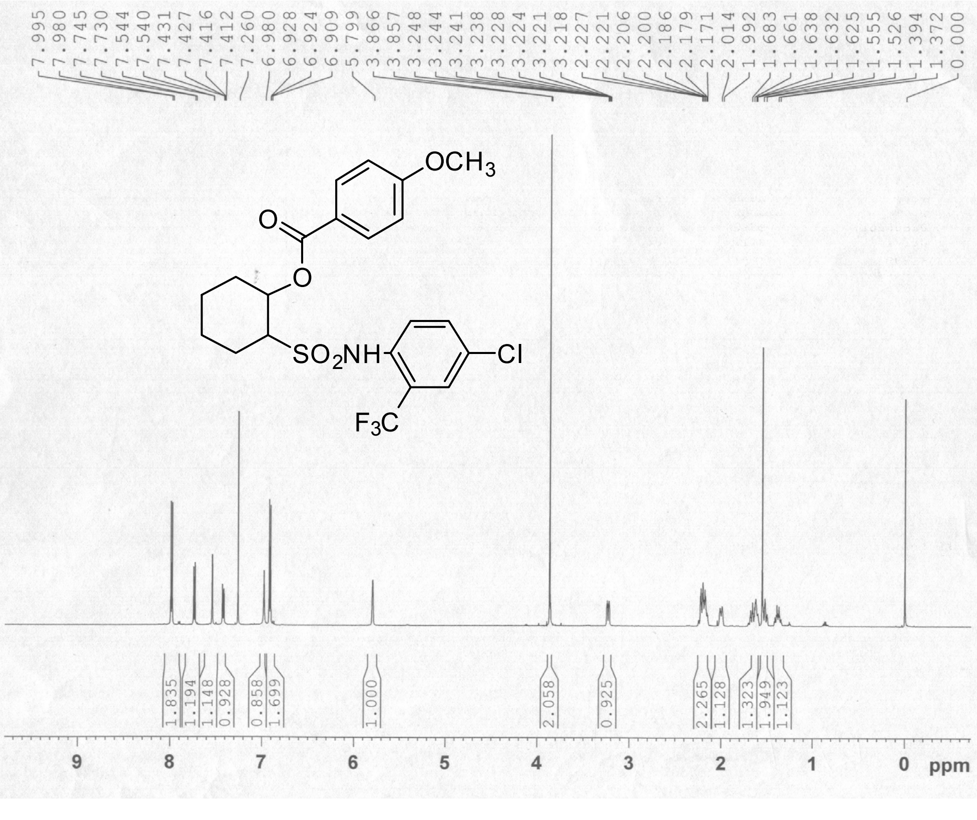

Figure S5. ^{1}H-NMR spectrum of compound **III-5**.

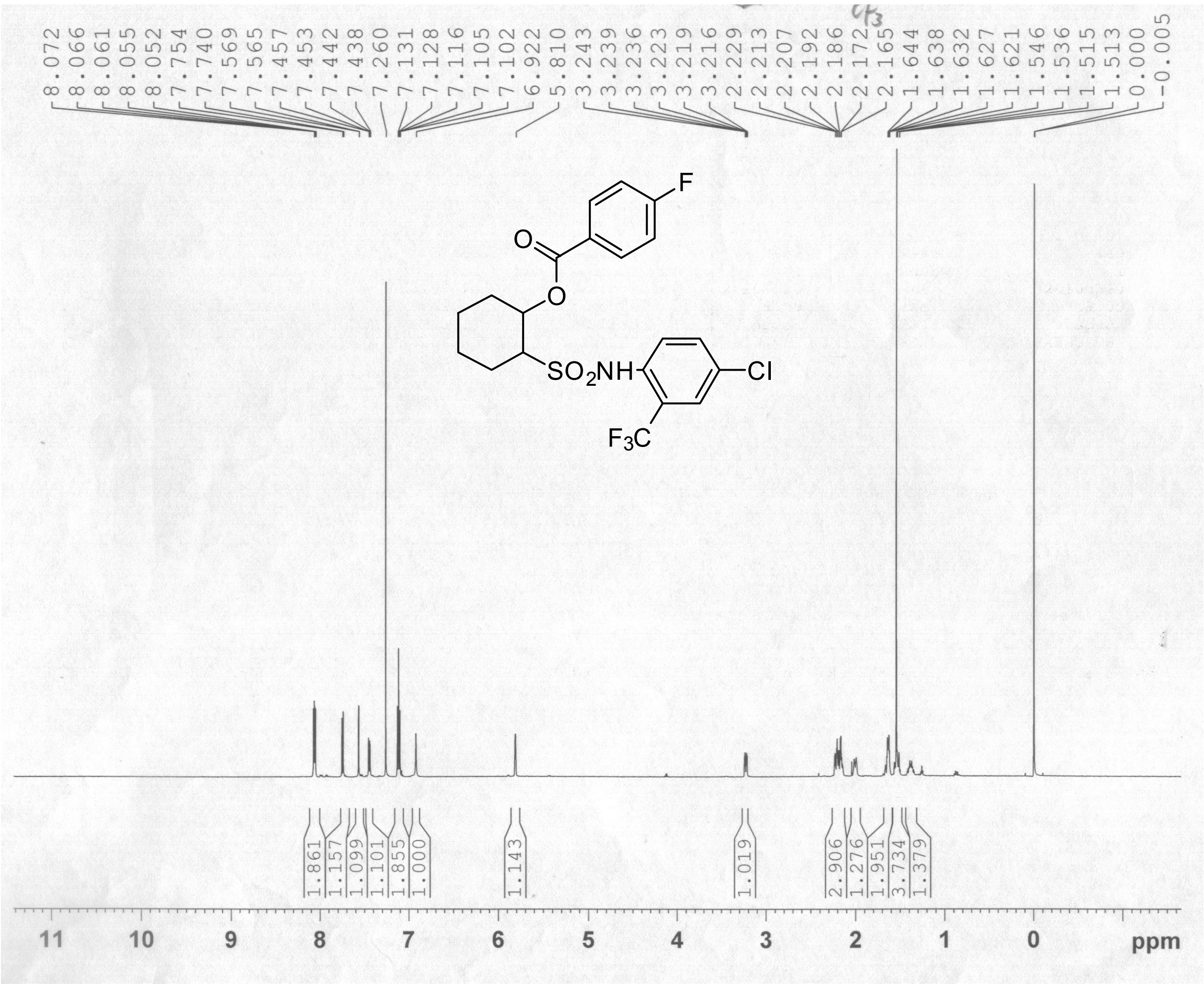

Figure S6. ^{1}H-NMR spectrum of compound **III-6**.

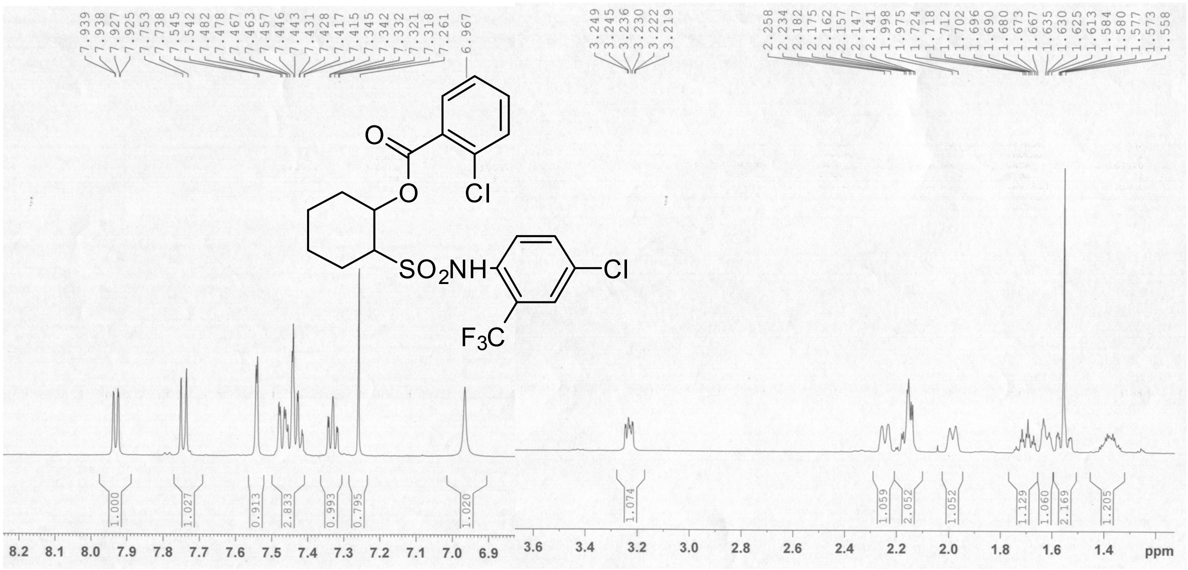

Figure S7. ^{1}H-NMR spectrum of compound **III-7**.

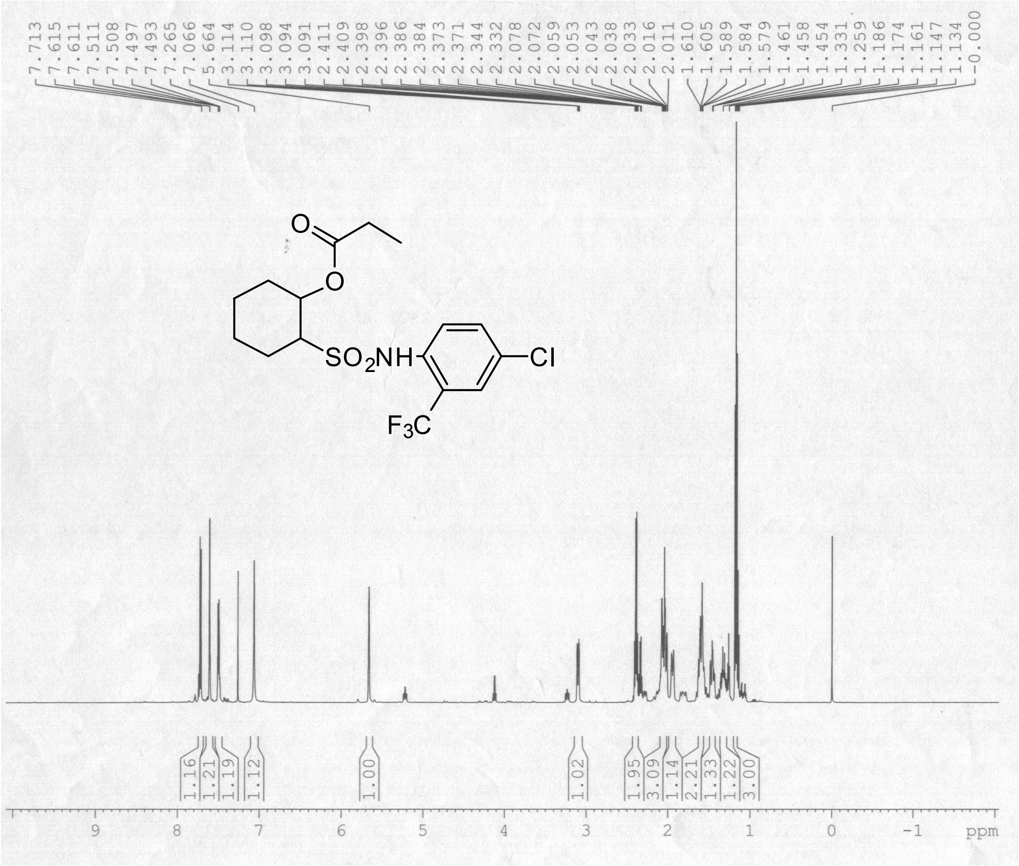

Figure S8. ^{1}H-NMR spectrum of compound **III-8**.

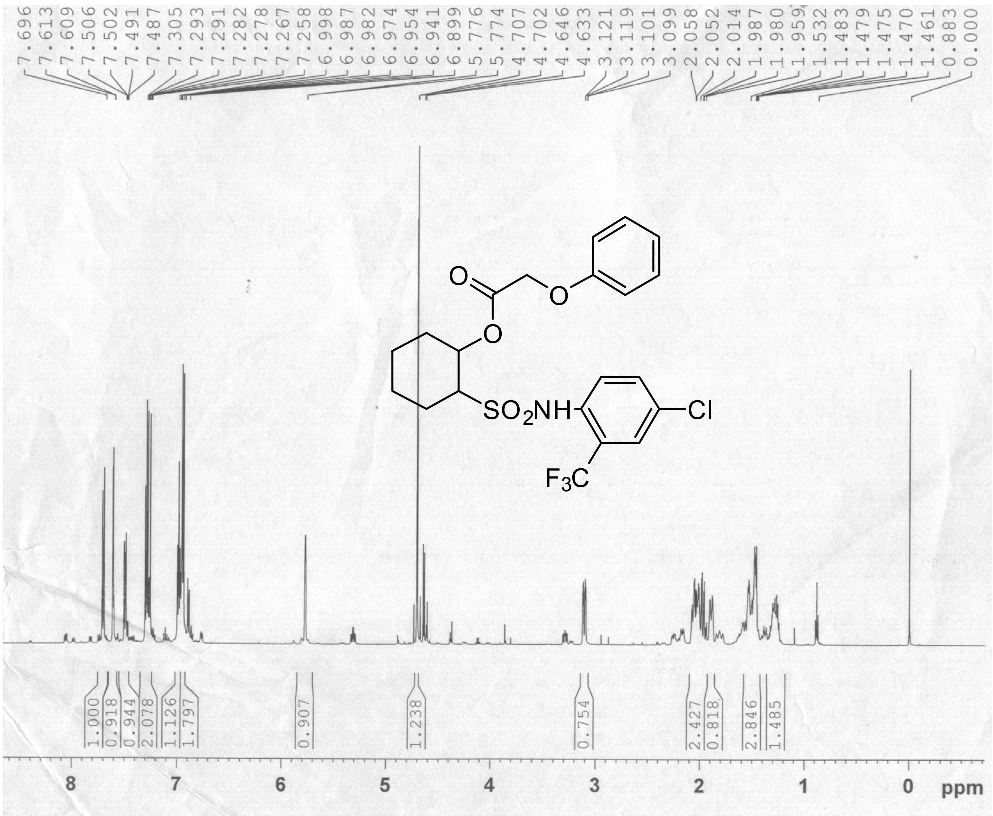

Figure S9. ^{1}H-NMR spectrum of compound **III-9**.

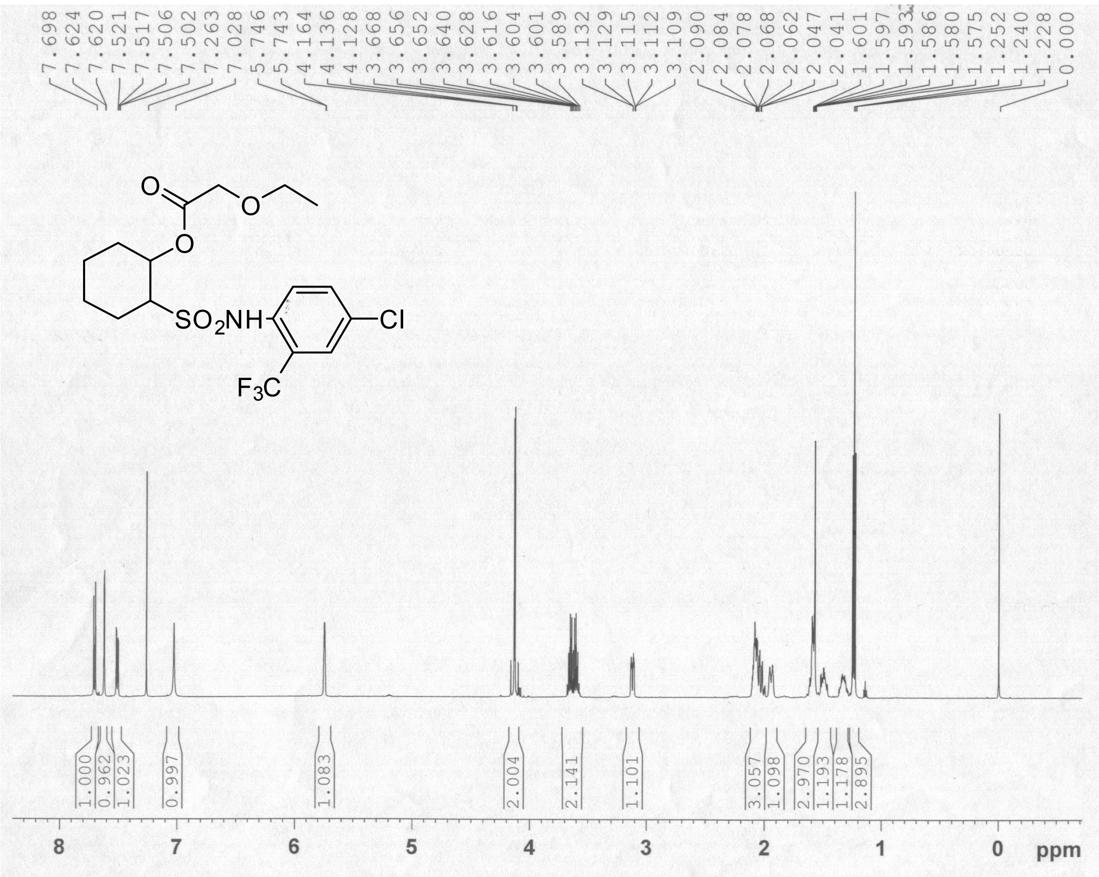

Figure S10. ^{1}H-NMR spectrum of compound **III-10**.

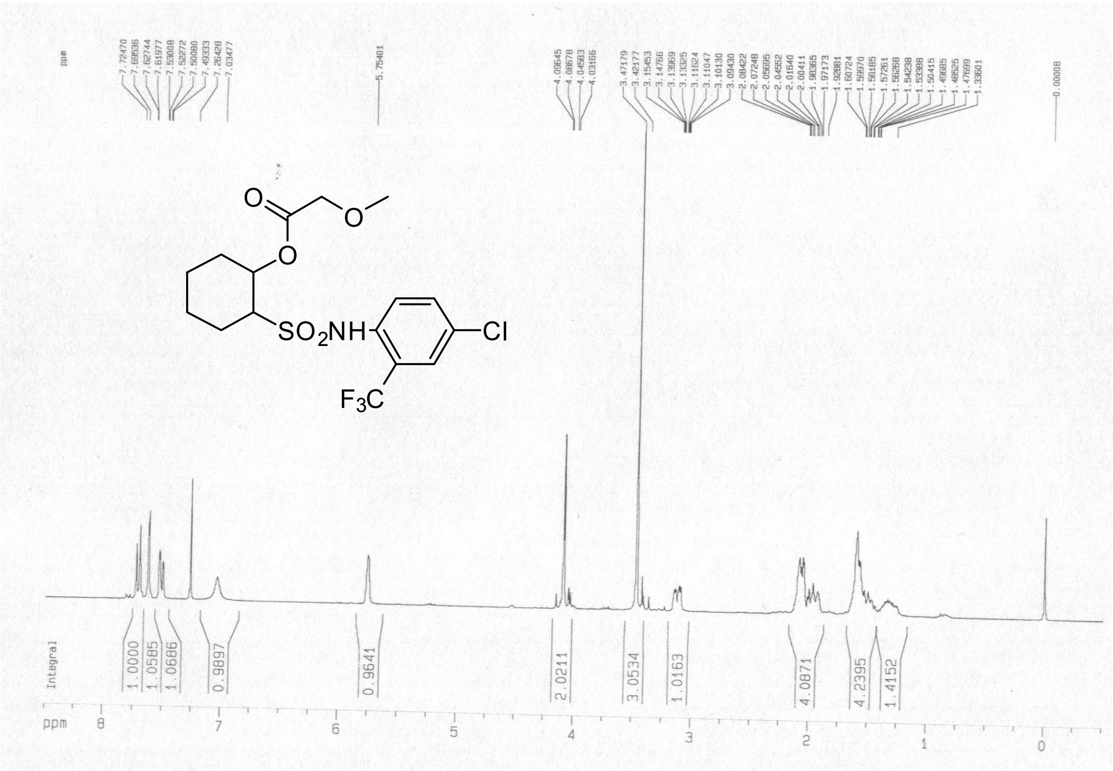

Figure S11. ^{1}H-NMR spectrum of compound **III-11**.

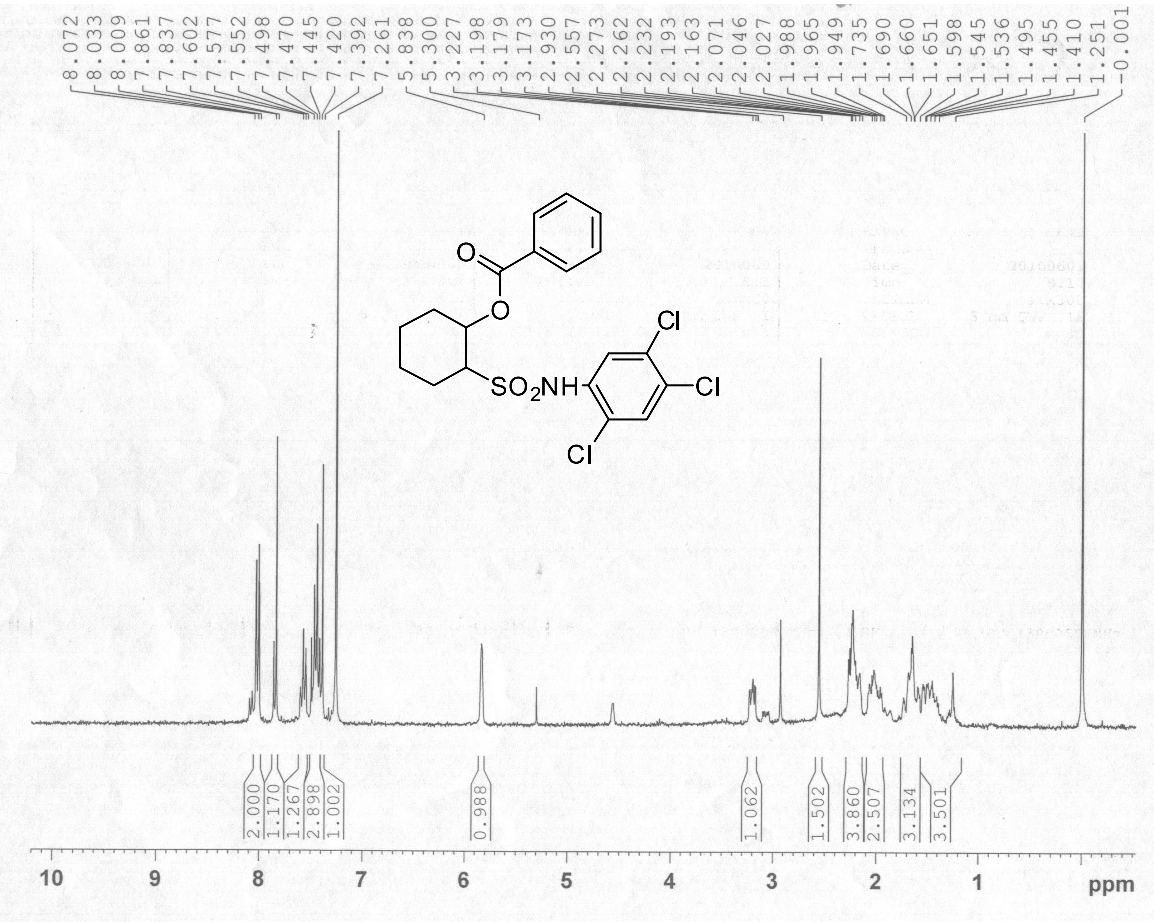

Figure S12. ^{1}H-NMR spectrum of compound **III-12**.

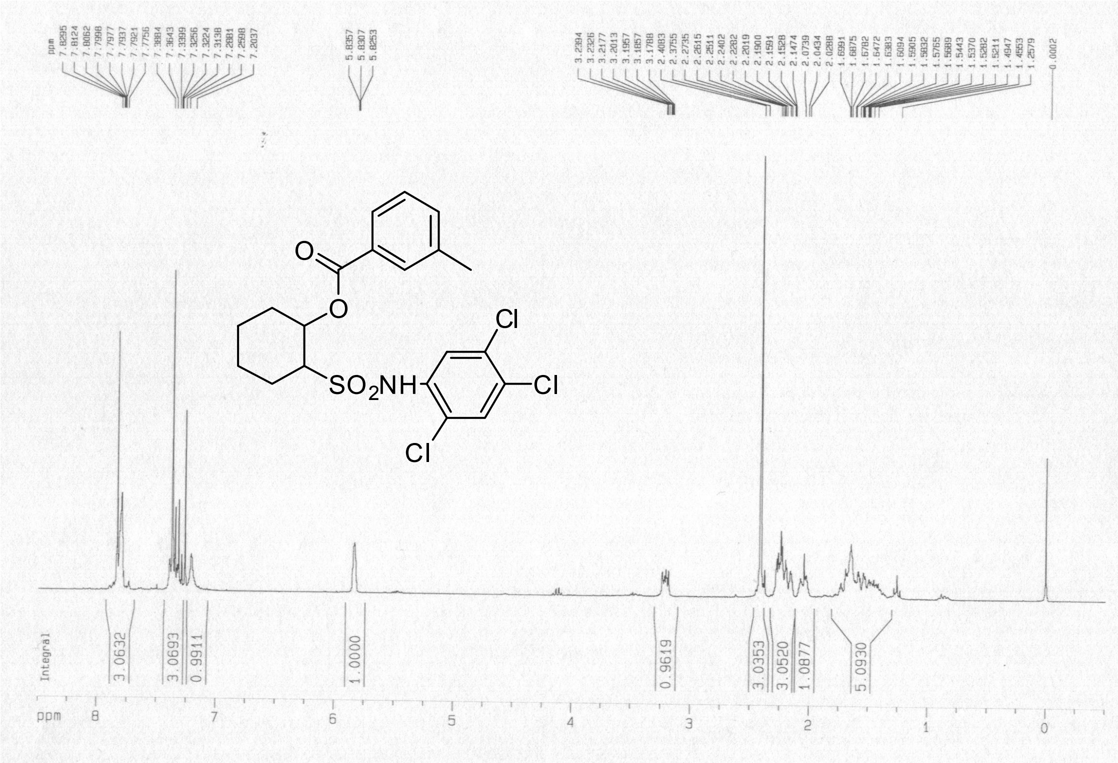

Figure S13. ^{1}H-NMR spectrum of compound **III-13**.

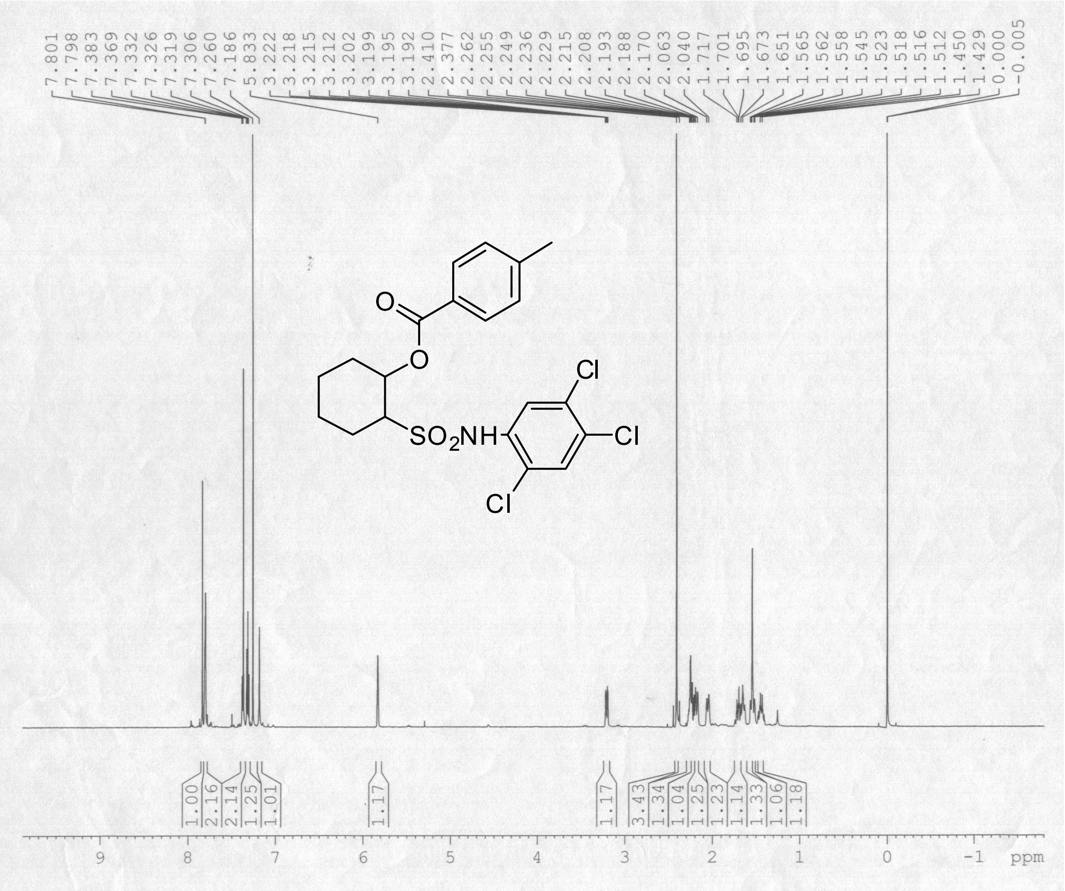

Figure S14. ^{1}H-NMR spectrum of compound **III-14**.

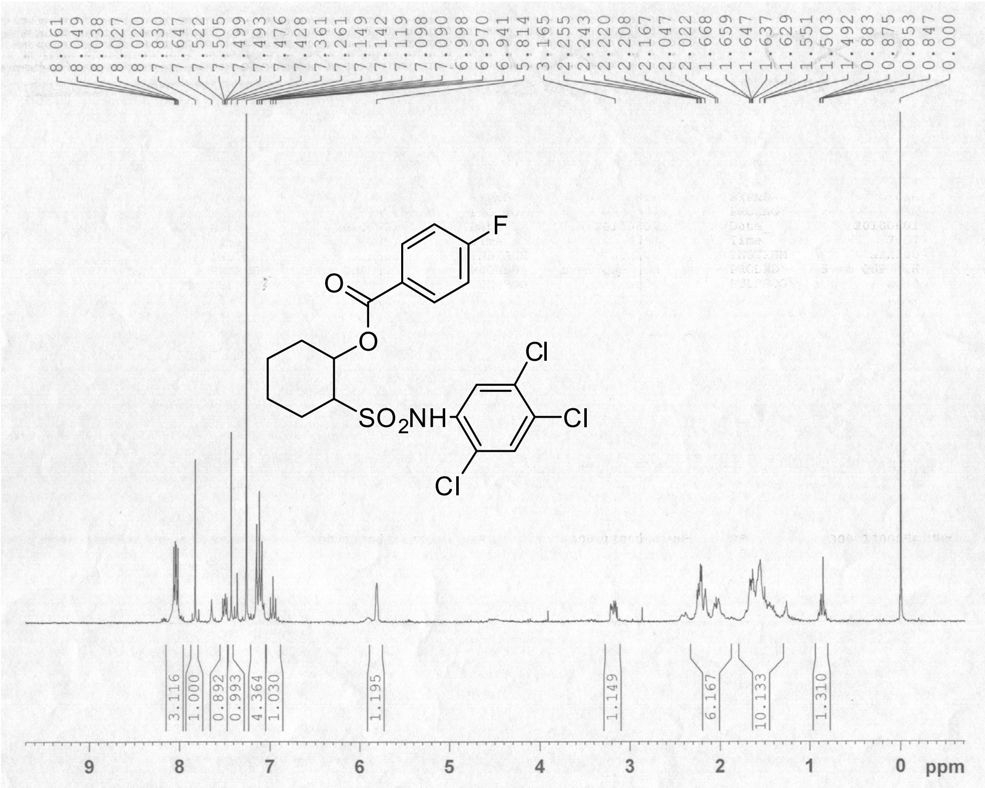

Figure S15. ^{1}H-NMR spectrum of compound **III-15**.

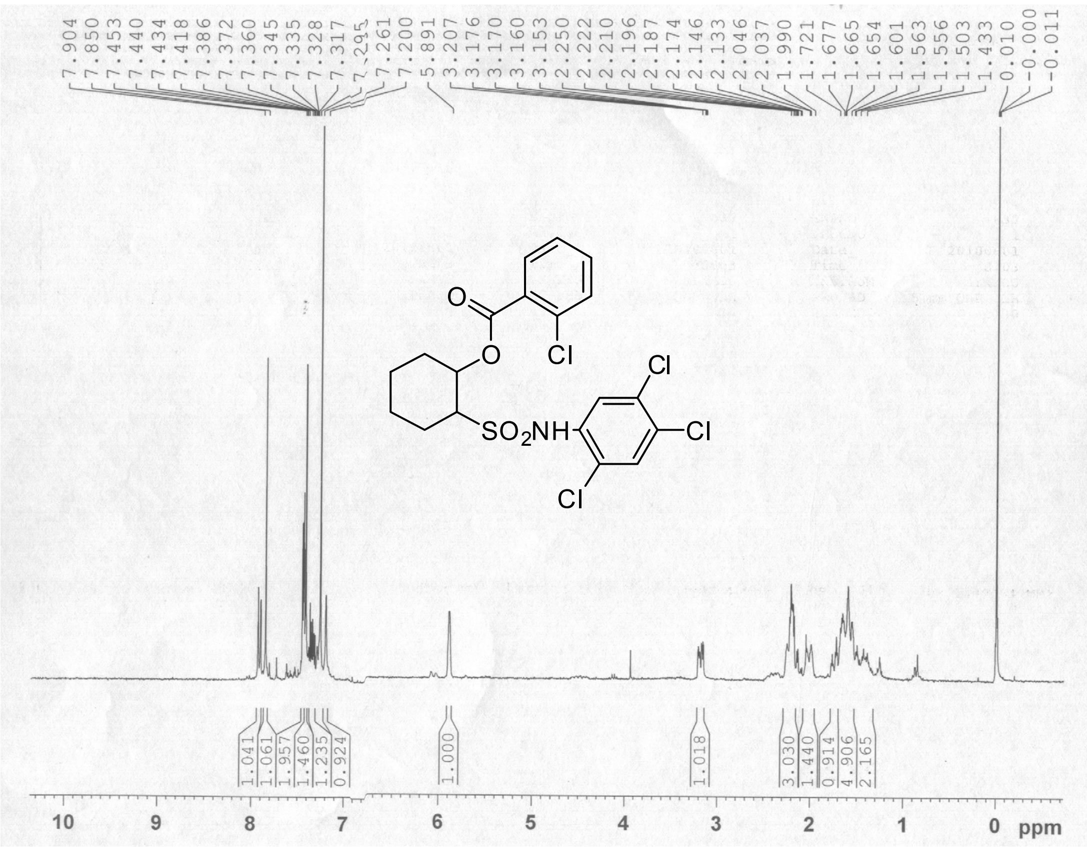

Figure S16. ^{1}H-NMR spectrum of compound **III-16**.

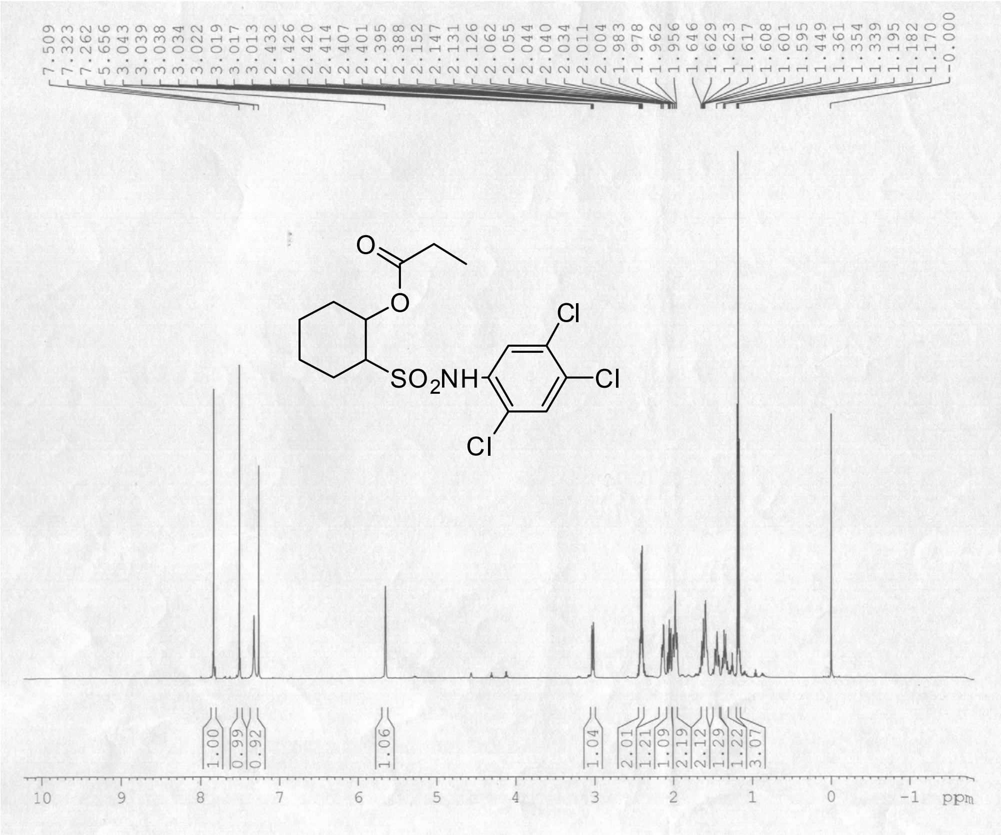

Figure S17. ^{1}H-NMR spectrum of compound **III-17**.

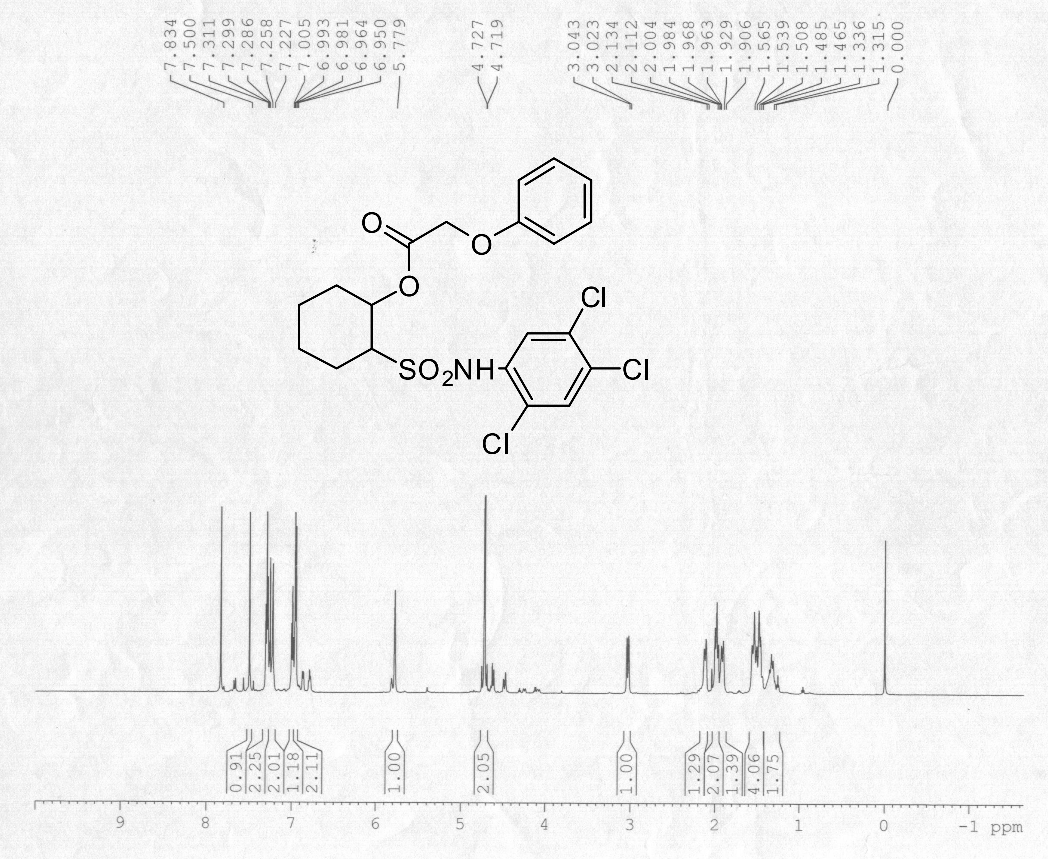

Figure S18. ^{1}H-NMR spectrum of compound **III-18**.

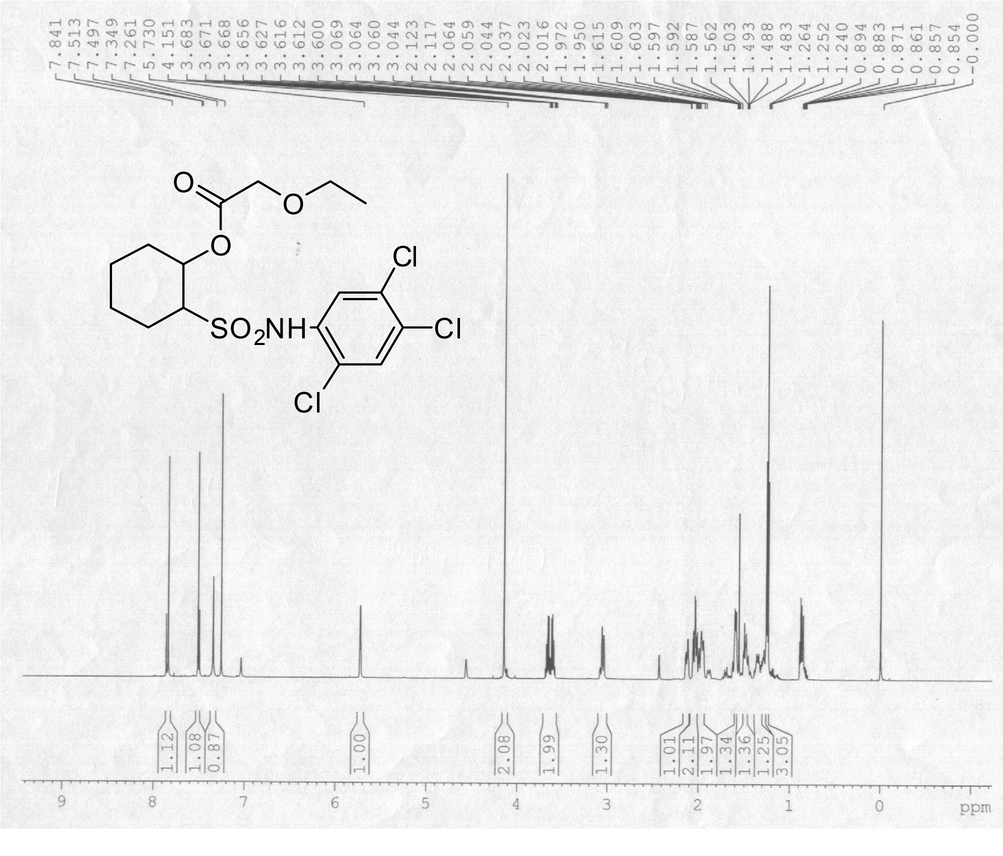

Supplement: Supplementary file 1 [file ijms-14-22544-s001.pdf]
